# Supplementary figures and images for: Morphine exposure exacerbates HIV-1 Tat driven changes to neuroinflammatory factors in cultured astrocytes
Source: PLoS One. 2020 Mar 25;15(3):e0230563. doi: 10.1371/journal.pone.0230563 (PMC7094849; doi:10.1371/journal.pone.0230563)

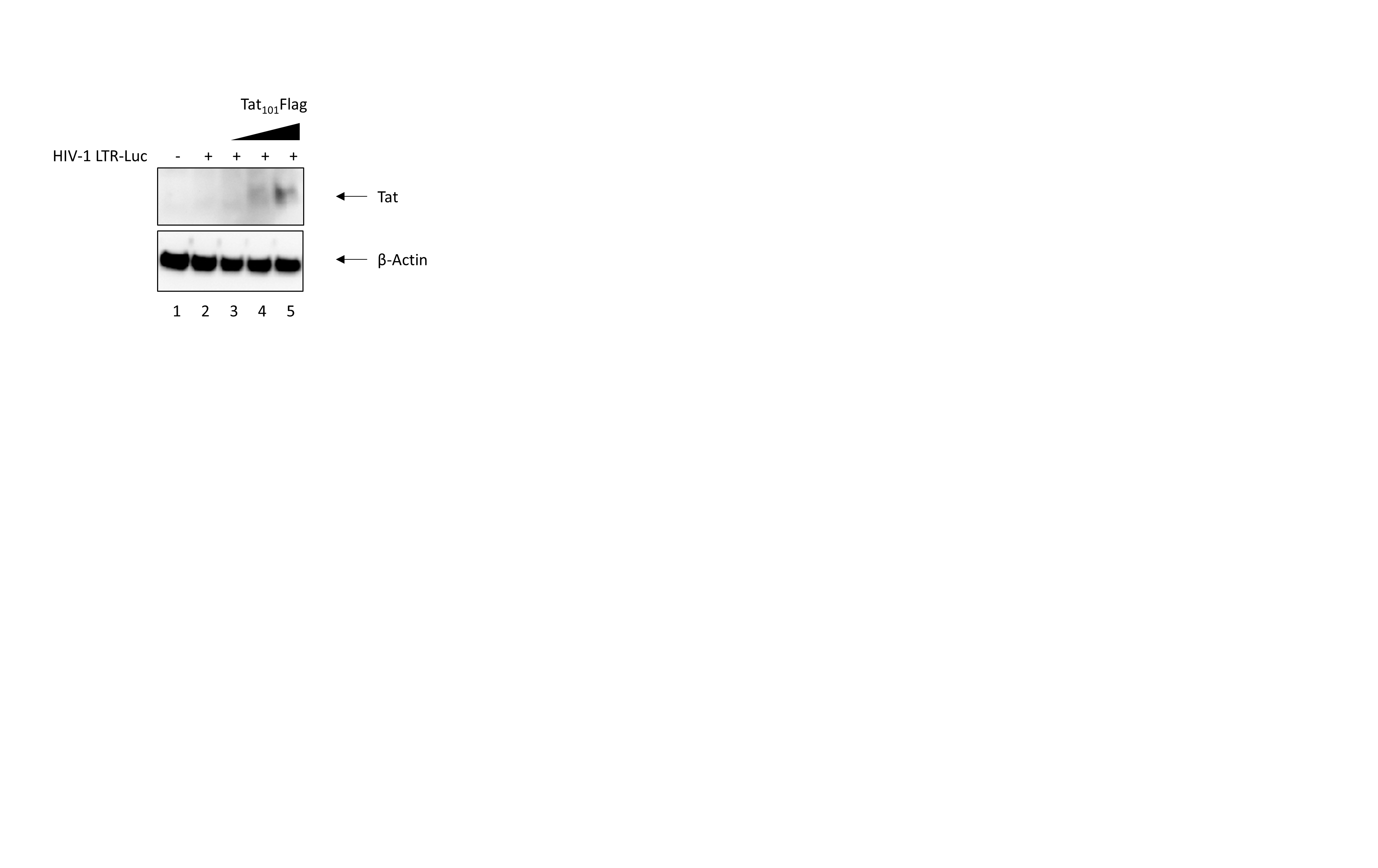

Supplement: S1 Fig — U87MG cells were transfected with an HIV-1 LTR-Luciferase reporter (pHIV-1-LTR-GL3; 1μg) in the presence or absence of increasing concentrations of a HIV-1 Tat expression vector (pCMV-Tat101-Flag; 8.4ng, 84ng, 840ng) corresponding to the low, medium and high conditions used. pUC19 transfection was included as a control. 48hrs post-transfection, cells were lysed and probed for HIV-1 Tat and β-Actin expression. (TIF) [file pone.0230563.s001.TIF]
